# 2384. Exploring COVID-19 Vaccine Hesitancy in Unvaccinated Adults within a Semi-Urban Population of South India: A Qualitative Investigation

**DOI:** 10.1093/ofid/ofad500.2004

**Published:** 2023-11-27

**Authors:** Dipu T Sathyapalan, Sivapriya Nair, Vrinda Nampoothiri, Merlin Moni

**Affiliations:** Amrita Hospital,Kochi, Ernakulam, Kerala, India; Amrita Hospital, Kochi, Ernakulam, Kerala, India; Amrita Hospital, Kochi, Ernakulam, Kerala, India; Amrita Institute of Medical Sciences, Kochi, Kochi, Kerala, India

## Abstract

**Background:**

This qualitative study examines COVID-19 vaccine hesitancy among unvaccinated adults in a semi-urban region of South India. Despite broad vaccination coverage in India, vaccine hesitancy persists, contributing to the ongoing transmission. By conducting region-specific in-depth qualitative research, we aim to identify barriers to vaccination, to address this public health menace.

**Methods:**

This grounded theory-based qualitative study explores COVID-19 vaccine hesitancy among unvaccinated adults in semi-urban regions of Ernakulam, Kerala, India. Conducted from April to May 2022, the study used purposive sampling to enrol participants with the help of ASHA workers. Semi-structured face-to-face interviews were carried out, utilizing an open-ended questionnaire-based interview guide. Audio files of the interviews were transcribed verbatim, and the data underwent a multistage thematic analysis using the constant comparative method.

**Results:**

In our study with 35 unvaccinated individuals, vaccine hesitancy factors were categorized into three themes: vaccine-related (33.33%), social/environmental (31.67%), and individual factors (35%). Individual factors included medical conditions (26.67%) and attitudes towards vaccines (8.33%). Vaccine-related factors involved safety concerns (15%), efficacy (6.67%), and administration mode (5%). Social/environmental factors covered accessibility/availability (16.67%) and influencers like healthcare workers, friends, and family (15%). Table 1, Figure 1, and Figure 2 provide further details on themes, subthemes, and their overlap.

Figure 1

**Figure:**
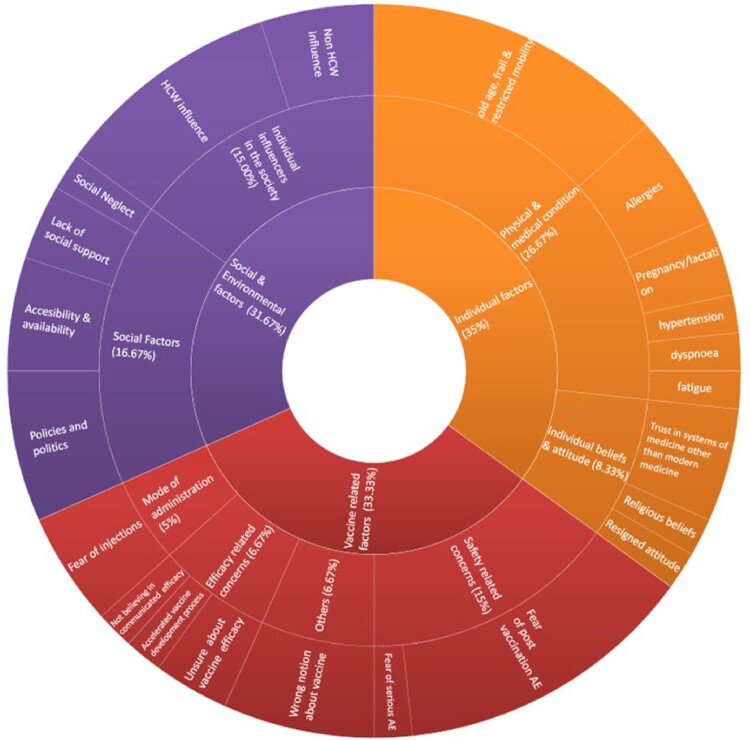

Representation of identified themes and subthemes among the vaccine hesitant individuals

Figure 2

**Figure:**
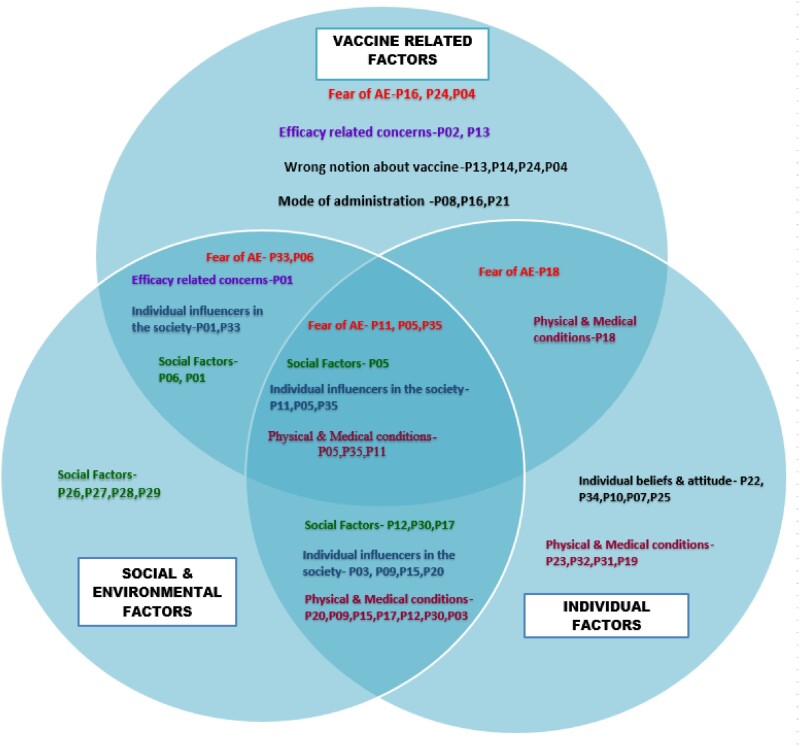

Schematic representation of identified themes and its overlap among the vaccine hesitant individuals.

Table 1

**Figure:**
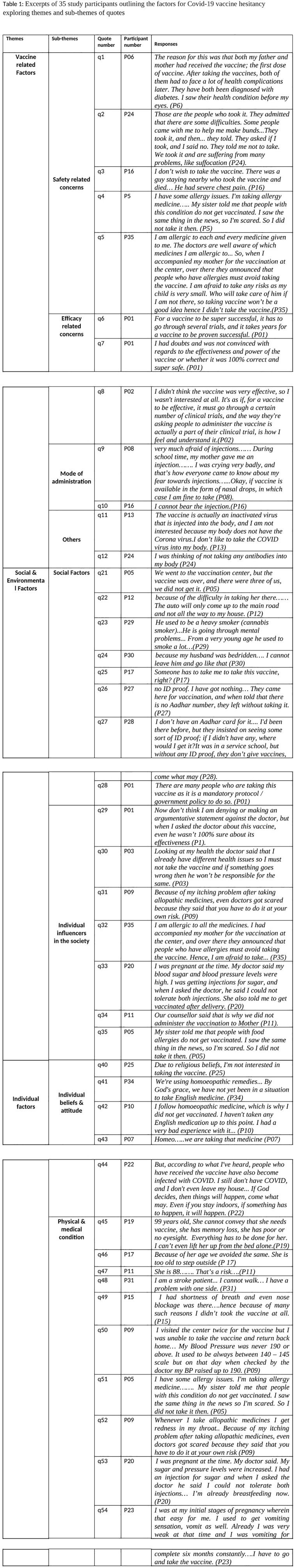

Excerpts of 35 study participants outlining the factors for Covid-19 vaccine hesitancy exploring themes and sub-themes of quotes

**Conclusion:**

Our study finds three main factors contributing to COVID-19 vaccine hesitancy: vaccine-related concerns, social/environmental influences, and individual factors. To tackle hesitancy, it's crucial to address safety concerns, engage with various medical systems, and leverage community influencers. Focusing on vaccine equity and addressing the needs of vulnerable populations is essential for the success of vaccination programs. Targeted communication strategies and behaviour change interventions should be employed to promote vaccine acceptance, and public health authorities must provide accurate, transparent information on vaccine safety and efficacy.

**Disclosures:**

**All Authors**: No reported disclosures

